# Implicit Motor Sequence Learning and Working Memory Performance Changes Across the Adult Life Span

**DOI:** 10.3389/fnagi.2016.00089

**Published:** 2016-04-26

**Authors:** Sarah Nadine Meissner, Ariane Keitel, Martin Südmeyer, Bettina Pollok

**Affiliations:** ^1^Institute of Clinical Neuroscience and Medical Psychology, Medical Faculty, Heinrich-Heine-UniversityDuesseldorf, Germany; ^2^Department of Neurology, Medical Faculty, Heinrich-Heine-UniversityDuesseldorf, Germany

**Keywords:** aging, SRTT, implicit motor sequence learning, consolidation, working memory, *n*-back

## Abstract

Although implicit motor sequence learning is rather well understood in young adults, effects of aging on this kind of learning are controversial. There is first evidence that working memory (WM) might play a role in implicit motor sequence learning in young adults as well as in adults above the age of 65. However, the knowledge about the development of these processes across the adult life span is rather limited. As the average age of our population continues to rise, a better understanding of age-related changes in motor sequence learning and potentially mediating cognitive processes takes on increasing significance. Therefore, we investigated aging effects on implicit motor sequence learning and WM. Sixty adults (18–71 years) completed verbal and visuospatial *n*-back tasks and were trained on a serial reaction time task (SRTT). Randomly varying trials served as control condition. To further assess consolidation indicated by off-line improvement and reduced susceptibility to interference, reaction times (RTs) were determined 1 h after initial learning. Young and older but not middle-aged adults showed motor sequence learning. Nine out of 20 older adults (compared to one young/one middle-aged) exhibited some evidence of sequence awareness. After 1 h, young and middle-aged adults showed off-line improvement. However, RT facilitation was not specific to sequence trials. Importantly, susceptibility to interference was reduced in young and older adults indicating the occurrence of consolidation. Although WM performance declined in older participants when load was high, it was not significantly related to sequence learning. The data reveal a decline in motor sequence learning in middle-aged but not in older adults. The use of explicit learning strategies in older adults might account for the latter result.

## Introduction

Implicit motor sequence learning refers to the ability to incidentally acquire knowledge of sequences of events and actions. The acquisition of such skills occurs “on-line” during practice but skills can stabilize—manifesting as reduced susceptibility to interference—or even improve “off-line” without further training (Robertson et al., 2004a, 2005). Reduced interference as well as off-line improvement constitute two components of the concept of consolidation (Robertson et al., 2004a). Previous studies suggest that consolidation requires an interval of at least 1 h after acquisition (Robertson et al., 2005; Janacsek and Nemeth, 2012). However, Pollok et al. (2014) who utilized the serial reaction time task (SRTT), a common paradigm to assess implicit motor sequence learning (Nissen and Bullemer, 1987), observed off-line changes after a break of only 10 min. Findings of how motor sequence learning changes with advancing age are controversial. Whereas some studies found intact acquisition in older adults (Howard and Howard, 1992; Brown et al., 2009; Nemeth and Janacsek, 2011), others suggest age-related declines in implicit motor sequence learning (Frensch and Miner, 1994; Howard et al., 2004). Two studies investigating sequence learning abilities not only in young and older adults but across the adult life span reported differing results. Gaillard et al. (2009) found no significant differences in motor sequence learning between young, middle-aged, and older adults, whereas Janacsek et al. (2012) reported a decrement in sequence learning abilities around the age of 45. Interestingly, changes of cortico-spinal interaction reflecting the integrity of the pyramidal’s system occur at this age as well (Kamp et al., 2013). Concerning consolidation of implicitly learned motor sequences in the elderly, studies are rare. There are a few studies reporting reduced or even lacking off-line improvement in healthy older adults when compared to younger ones (Spencer et al., 2007; Brown et al., 2009; Nemeth and Janacsek, 2011). To the best of our knowledge, age-related differences in susceptibility to interference, the second component of consolidation, have not been investigated directly so far.

Implicit motor sequence learning involves the striatum, the cerebellum as well as supplementary motor, primary motor, premotor and dorsolateral prefrontal cortices (DLPFC; Grafton et al., 1995; Destrebecqz et al., 2005; Doyon et al., 2009). Findings of whether these neural correlates change with advancing age are mixed. Daselaar et al. (2003), for example, found similar activations in young and older adults during implicit sequence learning, whereas others reported age-related changes in task-related activity with decreased activation in the DLPFC and striatum in older subjects (Aizenstein et al., 2006). Increased medial temporal lobe (MTL) activity might compensate for such changes in striatal structure and function with advancing age (Dennis and Cabeza, 2011).

Cognitive processes such as working memory (WM) seem to play an important role in motor sequence learning (Unsworth and Engle, 2005; Bo and Seidler, 2009). Regarding WM, which refers to the active storage and manipulation of information (Baddeley, 1992), evidence exists that performance declines with increasing age (Reuter-Lorenz et al., 2000; Park et al., 2002; for meta-analysis see Bopp and Verhaeghen, 2005). Studies using *n*-back paradigms in which participants were asked to indicate when the currently presented stimulus was the same as the one presented *n* trials back, reported poorer performance in the elderly than in younger subjects, especially when WM load was high (Mattay et al., 2006; Geerligs et al., 2014). Furthermore, it has been proposed that age-related declines in WM performance may contribute to age-related changes in motor sequence learning. This assumption has been supported by a study showing that older adults relied on WM processes to maximize learning performance (Bo et al., 2009). However, the authors used explicit motor learning tasks. Recently, Bo et al. (2011) provided the first evidence that in young adults, not only explicit but also implicit motor sequence learning is related to both verbal and visuospatial WM capacity, whereas in older adults verbal rather than visuospatial WM is suggested to be of importance to perform well in implicit motor sequence learning tasks (Bo et al., 2012). As is the case for motor sequence learning in general, the literature on potentially mediating cognitive processes is dominated by the comparison between young and older adults. Thus the understanding of changes across the adult life span is still sparse. At the neural level, a possible association between WM and motor sequence learning has at least partially been attributed to the suggested role of the DLPFC—a structure involved in WM processes (e.g., Jonides et al., 1993; D’Esposito et al., 1998; Curtis and D’Esposito, 2003)—in motor sequence learning (Bo et al., 2011). For example, disrupting normal functioning of the DLPFC by means of transcranial magnetic stimulation (TMS) impairs motor sequence learning in young adults (Pascual-Leone et al., 1996; Robertson et al., 2001).

As the average age of our population continues to rise, a better understanding of age-related changes in motor sequence learning and potentially mediating cognitive processes across the adult life span takes on increasing significance. In the present study, we aimed at investigating whether implicit motor sequence learning and consolidation as well as verbal and visuospatial WM changes across the adult life span and whether these processes are interrelated.

## Materials and Methods

### Participants

Twenty young (10 males, mean age: 23.65 ± 0.61 years [standard error of the mean; SEM], range: 18–29 years), 20 middle-aged (11 males, mean age: 36.25 ± 1.38 years, range: 30–50 years) and 20 older adults (10 males, mean age: 60.20 ± 1.50 years, range: 51–71 years) participated in the study. Exclusion criteria were: dementia (Mattis Dementia Rating Scale (MDRS) score ≤130; Mattis, 1988), history of neurological or psychiatric disorders, and medication affecting the central nervous system (CNS). Groups did not differ significantly with respect to mean years of education, visuospatial (Block-Tapping-Test; Schellis, 1997) or verbal short-term memory (Digit span; Von Aster et al., 2006; all *p* > 0.10). Although older adults scored significantly worse on the MDRS (median score 142.00) than young (median score 143.00; *U* = 1.93; *p* = 0.05) and middle-aged adults (median score 143.50; *U* = 2.06; *p* < 0.05), all participants scored within the normal range (score >130). All participants were right-handed as determined by the Edinburgh Handedness Inventory (Oldfield, 1971) and had normal or corrected-to-normal vision. The study was approved by the local ethics committee (study no. 4792) and was conducted according to the Declaration of Helsinki. All subjects participated voluntarily and provided written informed consent prior to the study.

### Testing Procedure and Tasks

**Figure 1:**
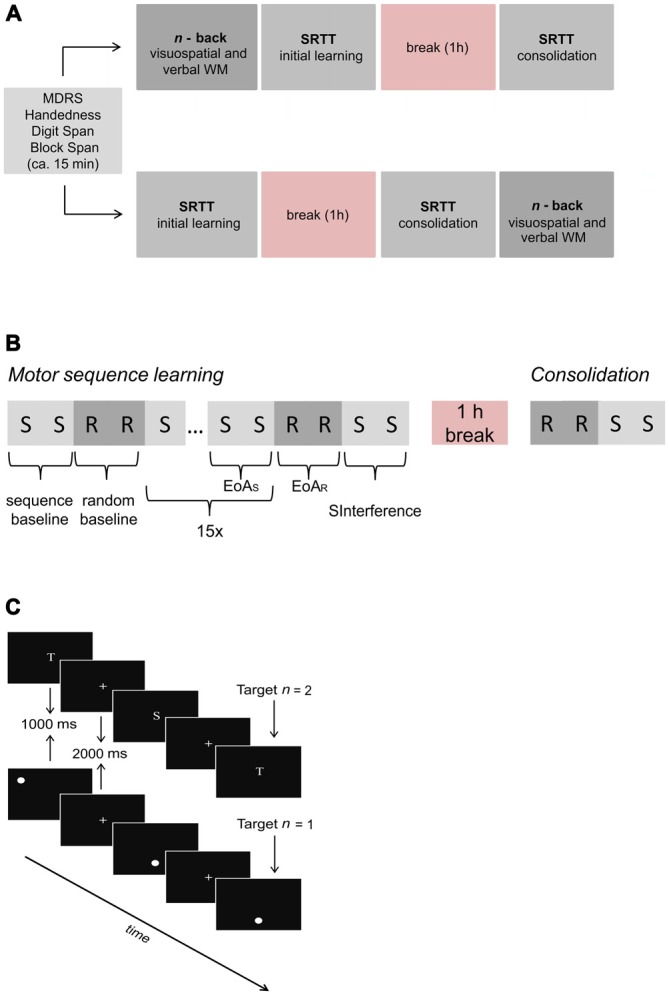
**Overview of the experimental procedure. (A)** Prior to serial reaction time task (SRTT) and *n*-back tasks, screening measures were assessed. Task order (SRTT vs. *n*-back) and task subtype (visuospatial vs. verbal *n*-back) were counterbalanced across subjects. **(B)** SRTT procedure. First, the sequence was introduced and presented twice (sequence baseline). After 16 randomly presented bars (random baseline), the sequence was presented 15 times with the last two sequences referred to as end of acquisition (EoA_S_). For the assessment of early interference, 16 randomly presented bars referred to as end of acquisition random (EoA_R_) were followed by two presentations of the sequence (SInterference). Run 2 (consolidation) consisted of 16 randomly presented bars which were followed by the presentation of the sequence (2 times). Note that “S” indicates sequence trials whereas “R” indicates random trials. **(C)** Sequence of events in three exemplary verbal 2-back (upper part) and visuospatial 1-back trials (lower part), respectively. Participants were asked to respond via button press whenever a stimulus occurred which was identical to the one presented “*n*” trials back.

To investigate visuospatial and verbal WM, motor sequence learning and potential links between these processes, all participants completed computerized verbal and visuospatial *n*-back tasks as well as the SRTT on the same day. Stimulus presentation and response recording were controlled by Eprime^®^ Software version 2 (Psychology Software Tools, Sharpsburg, PA, USA) installed on a standard windows computer. The order of task (SRTT vs. *n*-back) and task subtype (verbal vs. visuospatial *n*-back) was counterbalanced and randomly determined among participants within each group. Prior to performing the tasks, participants filled out handedness (Oldfield, 1971), biographical and health screening questionnaires and completed the MDRS (Mattis, 1988). Verbal and visuospatial short-term memory were assessed by means of the Block-Tapping-Test (Schellis, 1997) and the subtest “Digit Span” of the German version of the Wechsler Memory Scale Revised (Von Aster et al., 2006). Testing took approximately 2 h (1 h break included). For an overview of the experimental procedure, see Figure 1A.

#### Implicit Motor Sequence Learning: SRTT

The SRTT was introduced to the participants as a test of reaction time (RT). A custom-made response box with four response keys anatomically aligned to the right hand was used. Each response key corresponded to one of four horizontally aligned bars presented on a 19″ computer screen (1024 × 768 mm resolution; 75 Hz refresh rate). Participants were instructed to rest their thumb, index, middle and ring finger of their right hand on the response buttons and to press as quickly as possible the corresponding button as soon as one of the four bars on the computer screen changed from dark blue to light blue. RT was defined as the interval between the change in color and the button press onset. If participants responded correctly, the next bar was presented after a time interval of 1 s. In case of incorrect responses, the bar remained light blue until participants pressed the correct button.

Before starting the experimental phase, a practice block of 12 randomly presented bars was administered to familiarize participants with the response box. To assess motor sequence learning as well as consolidation, the task comprised two runs which were separated by a break of 1 h in which the subjects remained in the testing room without any specific task. The first two sequences of eight bar positions (*ring-index-thumb-middle-ring-middle-thumb-index*) were introduced and served as sequence baseline condition. The sequence used was a second-order conditional sequence that requires knowledge of the previous two positions to predict the next position, as the immediately preceding position alone provides insufficient information. Subjects were not informed of the existence of the sequence. Sixteen randomly presented bars serving as random baseline condition followed. To enable motor sequence learning, the same sequence as during sequence baseline was then repeated 15 times with the last two sequences referred to as end of acquisition (EoA_S_). Subsequently, 16 interfering randomly presented bars (EoA_R_) followed. To further examine whether the presentation of randomly presented bars interfered with the learned sequence immediately after learning (early interference), the sequence was again presented twice (SInterference). After a 1 h break, 16 randomly located bars followed by two repetitions of the previously learned sequence were presented in order to determine whether consolidation had occurred. Although implicit motor sequence learning is often investigated by sequences that consist of 10 or even 12 items we decided to use a sequence with only 8 bar positions. This decision was based on previous studies providing evidence that implicit motor sequence learning can be investigated by using an 8-item sequence, especially when the number of sequence presentations is rather low (Pollok et al., 2014, 2015; Krause et al., 2016). Figure 1B depicts the procedure of the SRTT.

Immediately after finishing the SRTT, participants were asked whether they had noticed anything significant or unusual about the task. If they were aware of the repeating pattern of the task, they were asked to recall the sequence.

#### WM: Verbal and Visuospatial *n*-back Tasks

The WM tasks were derived from the classical *n*-back task (Cohen et al., 1994) and required subjects to temporarily store and update information. Two subtypes (verbal vs. visuospatial), each with three WM loads (0- vs. 1- vs. 2-back) were used. Subjects were presented with a sequence of stimuli and were required to press a key whenever a presented stimulus was identical to the one presented *n* trials back. The 0-back tasks did not involve WM and were used as attentional control tasks (Cohen et al., 1994). In each task, participants were asked to respond as accurately and quickly as possible.

In the verbal 1-, and 2-back tasks, stimuli consisted of six white capital letters (N, I, R, S, T, A; see Figure 1C) which appeared serially and pseudorandomly on a black background in the center of the computer screen. The letters were selected from the lexical database dlexDB © (dlex project, DWDS Project, University of Potsdam, Germany) and matched with respect to frequency of occurrence. Each letter was presented for 1 s and was followed by a white central fixation cross presented for 2 s. Participants were asked to press the right arrow button on a conventional computer keyboard every time the currently presented letter matched the letter presented one (1-back) or two trials (2-back) back. Responses occurring after 3 s were coded as “miss”. In case of a non-match, subjects did not need to press any button. In the 0-back task, the letter “X” was presented in addition to the six other letters and participants were required to press the right arrow button every time an “X” appeared on the screen.

In the visuospatial 1-, and 2-back tasks, stimuli consisted of identical white circles with a diameter of 1.75 cm which appeared serially and pseudorandomly for 1 s in one of six possible locations on the black screen. They were followed by the presentation of a white central fixation cross for 2 s (see Figure 1C). The six locations corresponded to the center of the cells of a 2 × 3 grid with the size of 12.75 cm by 13.50 cm. During the experiment, grid lines were not presented. Participants were asked to press the right arrow button on the computer keyboard every time the circle appeared in the same location as the circle one (1-back) or two trials (2-back) back. In case of a non-match, subjects did not need to press any button. In the 0-back task, participants were required to press the right arrow button every time the circle appeared in the center of the screen, a location engaged in this condition only.

A brief practice sequence of 10 trials was given prior to each task which consisted of five matching letters or locations with 17, 18 and 19 stimuli presented in the 0-, 1-, and 2-back tasks, respectively. While subtype order (verbal vs. visuospatial) was counterbalanced across participants, order of WM load was fixed and increased from low to high (0-, 1-, 2-back).

### Data Analyses

#### SRTT

Mean RTs for baseline, EoA, and consolidation trials for sequence and random trials separately as well as a mean RT for early interference sequence trials were calculated. RTs two standard deviations below or above the respective individual mean as well as the group mean were excluded from further analyses. Furthermore, the percentage of errors was calculated for each participant.

#### WM Tasks

Performance in each *n*-back task was quantified as percentage of hits (correct responses and correct non-responses) in individual data for each task subtype and load. One older adult was excluded from further analyses as she did not understand task instructions, leaving a final sample of *n* = 19 for the older group.

### Statistical Analyses

Statistical analyses were performed using IBM SPSS 22 (IBM Corporation, Armonk, NY, USA). All tests for statistical significance were two-sided. For the SRTT, Kolmogorov-Smirnov tests did not show evidence that the data significantly deviate from Gaussian distribution. Therefore, mixed-design analysis of variance (ANOVA) on mean RT with *time* (baseline vs. EoA) and *condition* (sequence vs. random) as within-subjects factors and *group* (young vs. middle-aged vs. older adults) as between-subjects factor was conducted to assess motor sequence learning. In order to compare learning effects more directly and to control for possible baseline RT differences between groups, we investigated percentage RT improvements. Each participant’s gain in RT during sequence trials (sequence baseline—EoA_S_) was divided by the respective sequence baseline RT and multiplied by 100. The same score was calculated for gain in RT during random trials ((random baseline—EoA_R_)/random baseline × 100). Participants with percentage RT improvements two standard deviations below or above the respective group mean were excluded from further analyses. The scores were subjected to a mixed-design ANOVA with *condition* (sequence vs. random) and *group* as factors. To assess early susceptibility to interference immediately after learning, mixed-design ANOVA on mean RT with the factors *time* (EoA_S_ vs. SInterference) and *group* was conducted. The investigation of consolidation was realized with two mixed-design ANOVAs on mean RT. For susceptibility to interference, we performed an ANOVA with the factors *group* and *time* (SInterference vs. sequence consolidation). To estimate off-line improvement, an ANOVA with *time* (EoA vs. consolidation), *condition* (random vs. sequence) and *group* was conducted. Potential differences in mean percentage of errors between the three age groups were investigated by a one-way ANOVA. To resolve interactions, *post hoc* tests were calculated by means of two-tailed *t*-tests and ANOVAs.

For WM data, Kolmogorov-Smirnov tests revealed deviations from normal distribution requiring nonparametric tests. We examined whether verbal and visuospatial 0-, 1-, and 2-back WM performance differed in any of the age groups using Wilcoxon signed-rank tests. As none of the tests revealed significant differences between the two subtypes in any of the groups (all *p* > 0.25), percentage of hits for verbal and visuospatial subtypes were pooled for 0-, 1-, and 2-back tasks, respectively. Kruskal-Wallis tests were applied to determine WM differences between age groups in the 0-, 1-, and 2-back tasks, respectively.

Spearman correlations were calculated to examine whether and to what extent motor sequence learning and consolidation is related to WM performance. To this end, we calculated a sequence learning (sequence baseline—EoA_S_) as well as a random control score (random baseline—EoA_R_), an early interference (EoA_S_—SInterference) and consolidation scores (SInterference—sequence consolidation; EoA_S_—sequence consolidation) for each individual. Each of these scores was correlated with the pooled percentage of hits for verbal and visuospatial subtypes in 1- and 2-back tasks, respectively. To further investigate whether WM is related to RTs in general, percentage of hits in 1- and 2-back tasks respectively was also correlated with baseline RTs. All correlations were calculated for different age groups separately as well as for the entire group. Bonferroni corrections for multiple testing were applied.

## Results

Due to RTs two standard deviations below or above the respective individual mean, max 6.02% of trials in young and 6.48% of trials in middle-aged and older adults respectively were excluded from further SRTT analyses. At the group level, two participants of each age group showed RTs that were more than two standard deviations below or above the respective group mean in baseline, EoA and interference trials and were thus excluded from motor sequence learning and susceptibility to interference analyses. For similar reasons, two young and two older as well as three middle-aged adults were excluded from off-line improvement analysis. Concerning percentage RT improvement, one young, three middle-aged and two older adults were excluded from analysis due to gains in RT that were more than two standard deviations below or above the respective group mean.

### Motor Sequence Learning

**Figure 2:**
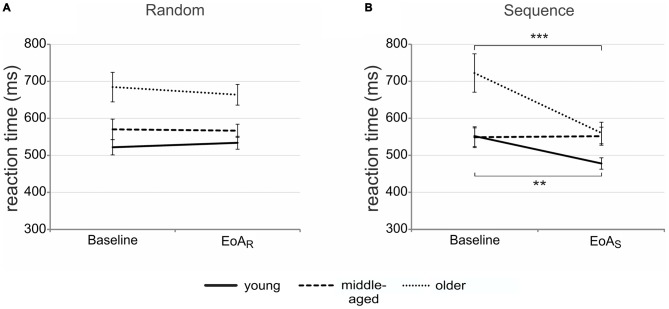
**Motor sequence learning.** Mean reaction times (RTs) for randomly **(A)** and sequentially **(B)** presented bars at baseline and end of acquisition in the three age groups. Error bars indicate standard error of the mean (SEM); **p < 0.01; ***p < 0.001; end of acquisition random (EoA_R_); end of acquisition sequence (EoA_S_).

Since we were interested in the effect of age on motor sequence learning, only main effects or interactions involving the factor *group* are reported here. Mixed-design ANOVA with *time* (baseline vs. EoA), *condition* (sequence vs. random) and *group* (young vs. middle-aged vs. older adults) as factors revealed a significant main effect of *group* [*F*_(2,5)_ = 7.78; *p* = 0.001; $\eta_{p}^{2}$ = 0.23] with *post hoc* *t*-tests indicating slower RTs in older than in young [*t*_(34)_ = 3.46; *p* = 0.001] and middle-aged adults [*t*_(34)_ = 2.47; *p* = 0.02] regardless of condition and time. Importantly, a significant *group* by *time* [*F*_(2,51)_ = 4.33; *p* = 0.02; $\eta_{p}^{2}$ = 0.15] and *group* by *condition* by *time* interaction emerged [*F*_(2,51)_ = 10.19; *p* < 0.001; $\eta_{p}^{2}$ = 0.29; see Figures 2A,B].

**Figure 3:**
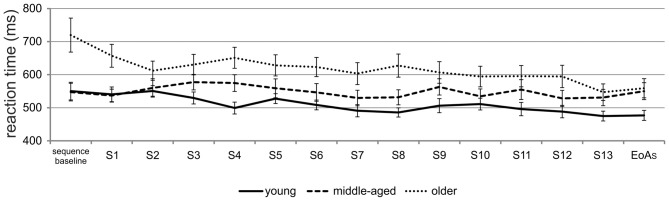
**Learning curves for all age groups.** Mean RTs during training on the SRTT in young, middle-aged and older adults; end of acquisition sequence (EoA_S_); S = sequence (e.g., S1 = sequence 1).

To resolve the three-way interaction, *post hoc* ANOVAs were conducted. Separate ANOVAs for the factor *group* revealed a significant interaction between *condition* and *time* for young and older [young: *F*_(1,17)_ = 36.59; *p* < 0.001; $\eta_{p}^{2}$ = 0.68; older: *F*_(1,17)_ = 33.04; *p* < 0.001; $\eta_{p}^{2}$ = 0.66] but not for middle-aged adults [*F*_(1,17)_ = 0.05; *p* = 0.83; $\eta_{p}^{2}$ = 0.003]. In the random condition, RTs between baseline and EoA_R_ trials did not differ significantly, neither in older nor in young adults (all *p* > 0.36). When the sequence was presented, however, both young and older adults were significantly faster at EoA_S_ as compared to baseline [young: *t*_(17)_ = 3.58; *p* = 0.002; older: *t*_(17)_ = 5.43; *p* < 0.001]. The data suggest that motor sequence learning occurred in young and older but not in middle-aged participants. *Post hoc* *t*-tests for independent samples revealed that at sequence baseline, older adults were significantly slower than young [*t*_(34)_ = 2.93; *p* = 0.006] and middle-aged adults [*t*_(34)_ = 2.98; *p* = 0.005]. However, at EoA_S_, both middle-aged [*t*_(34)_ = 2.55; *p* = 0.02] and older adults [*t*_(34)_ = 2.22; *p* = 0.03] were significantly slower than young ones. To further exclude the possibility that middle-aged adults showed motor sequence learning in earlier phases of the task, we calculated the mean RT for each sequence during learning and selected the sequence with the fastest RT (sequence 12, see learning curves presented in Figure 3). However, the comparison with sequence baseline RT did not result in significant differences (*p* = 0.50), ruling out the possibility that middle-aged adults showed motor sequence learning earlier in the course of the SRTT.

Mixed-design ANOVA with *condition* (sequence vs. random) and *group* (young vs. middle-aged vs. older adults) as factors which was conducted to investigate percentage gains in SRTT performance revealed a significant main effect of *group* [*F*_(2,51)_ = 5.94; *p* = 0.005; $\eta_{p}^{2}$ = 0.19] with *post hoc* *t*-tests indicating higher RT gains in older than in young [*t*_(35)_ = 2.56; *p =* 0.04] and middle-aged adults [*t*_(33)_ = 3.48; *p* = 0.006]. A significant *group* by *condition* interaction [*F*_(2,51)_ = 12.65; *p* < 0.001; $\eta_{p}^{2}$ = 0.33] revealed significantly greater gains in RT in sequence than in random trials in young and older [young: sequence mean = 10.63 ± 12.45 [SD]; random mean = −3.46 ± 12.55; *t*_(18)_ = 5.50; *p* < 0.001; older: sequence mean = 21.87 ± 11.14; random mean = 2.41 ± 12.03;* t*_(17)_ = 5.57; *p* < 0.001] but not in middle-aged adults (sequence mean = −2.06 ± 14.83; random mean = 3.63 ± 13.91; *t*_(16)_ = −1.18; *p* = 0.26). Interestingly, for sequence trials, *post hoc* *t*-tests for independent samples revealed not only lower percentage RT improvements in middle-aged adults when compared to young [*t*_(34)_ = −2.79; *p* = 0.009] and older ones [*t*_(33)_ = 5.42; *p* < 0.001] but also lower percentage RT improvements in young than in older adults [*t*_(35)_ = 2.89; *p* = 0.007]. There was no significant difference between groups when random scores were compared (all *p* > 0.12).

### Susceptibility to Interference and Off-Line Improvement

**Figure 4:**
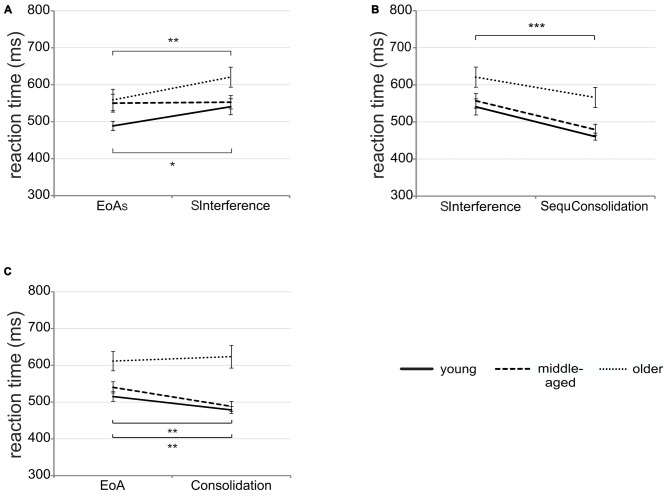
**Early interference and off-line changes. (A)** Mean RTs in the three age groups on sequence trials (SInterference) after 16 interfering randomly presented bars as compared to sequence trials at end of acquisition (EoA_S_). **(B)** Mean RTs in the three age groups on sequence trials after 1 h break (SequConsolidation) as compared to sequence interference trials (SInterference). **(C)** Significant *time* by *group* interaction showing mean RTs in the three age groups on consolidation trials after 1 h break as compared to trials at EoA. Note that consolidation and EoA trials refer to pooled sequence and random trials. Error bars represent SEM; *p < 0.05; **p < 0.01; ***p < 0.001.

To examine potential differences between age groups in susceptibility to interference immediately after learning, we conducted mixed-design ANOVA with *time* (EoA_S_ vs. SInterference) and *group* (young vs. middle-aged vs. older adults) as factors. The main effect of *group* tended to be significant [*F*_(2,51)_ = 3.16; *p* = 0.05; $\eta_{p}^{2}$ = 0.11] with faster RTs in young than in older adults [*t*_(34)_ = 2.46; *p* = 0.04]. Young and middle-aged [*t*_(34)_ = 1.46; *p* = 0.15) as well as middle-aged and older adults [*t*_(34)_ = 1.15; *p* = 0.26] did not differ significantly. In addition, a significant *time* by *group* interaction emerged [*F*_(2,51)_ = 3.42; *p* = 0.04; $\eta_{p}^{2}$ = 0.12; see Figure 4A]. *Post hoc* *t*-tests revealed that in young and older adults, RTs were significantly faster at EoA_S_ than at SInterference (young: *t*_(17)_ = 2.88; *p* = 0.01; older: *t*_(17)_ = 3.33; *p* = 0.004) indicating susceptibility to interference in both groups. In middle-aged participants, no significant difference emerged [*t*_(17)_ = 0.16; *p* > 0.87].

The analysis of potential differences between age groups in susceptibility to interference 1 h after initial learning revealed a significant main effect of *group* [*F*_(2,51)_ = 6.93; *p* = 0.002; $\eta_{p}^{2}$ = 0.21] with faster RTs in young and middle-aged than in older adults [young vs. older adults: *t*_(34)_ = 3.26; *p* = 0.003; middle-aged vs. older adults: *t*_(34)_ = 2.53; *p* = 0.02] while no significant difference between young and middle-aged adults emerged [*t*_(34)_ = 0.87; *p* = 0.39]. The significant main effect of *time* [*F*_(1,51)_ = 42.37; *p* < 0.001; $\eta_{p}^{2}$ = 0.45; see Figure 4B] suggests faster RTs in consolidation than in early interference trials. The *time* by *group* interaction failed to reach significance [*F*_(2,51)_ = 0.55; *p* = 0.58; $\eta_{p}^{2}$ = 0.02].

The ANOVA conducted to investigate off-line improvement revealed a significant main effect of *group* [*F*_(2,50)_ = 12.66; *p* < 0.001; $\eta_{p}^{2}$ = 0.33] with faster RTs in young and middle-aged than in older adults [young vs. older adults: *t*_(34)_ = 4.16; *p* < 0.001; middle-aged vs. older adults: *t*_(33)_ = 3.39; *p* = 0.001] but no significant difference between young and middle-aged adults [*t*_(33)_ = 1.01; *p* = 0.32]. Furthermore, we found a significant *condition* by* group* interaction [*F*_(2,50)_ = 10.34; *p* < 0.001; $\eta_{p}^{2}$ = 0.29]. *Post hoc* *t*-tests revealed significantly faster RTs in sequence than in random trials in young and older [young: *t*_(17)_ = 6.25; *p* < 0.001; older: *t*_(17)_ = 6.53; *p* < 0.001] but not in middle-aged adults [*t*_(16)_ = 2.04; *p* = 0.06]. Moreover, the *time* by *group* interaction reached significance [*F*_(2,50)_ = 4.89; *p* = 0.01; $\eta_{p}^{2}$ = 0.16; see Figure 4C]. *Post hoc* *t*-tests revealed significantly faster RTs in consolidation trials than at EoA for both young [*t*_(17)_ = 3.50; *p* = 0.003] and middle-aged adults [*t*_(16)_ = 3.64; *p* = 0.002]. In older participants, RTs at EoA and consolidation did not differ significantly [*t*_(17)_ = −0.66; *p* = 0.52] suggesting no off-line improvement in this group. As the *time* by *condition* by *group* interaction did not reach significance (*p* = 0.53) the findings indicate unspecific RT facilitation over the off-line period.

### SRTT Error Rates

As expected for a SRTT, mean percentages of errors were low (overall mean: 2.71 ± 2.61 [SD]; young: 2.50 ± 2.20; middle-aged: 2.89 ± 2.95; older: 2.73 ± 2.72) and did not differ between age groups (*p* = 0.89).

### Awareness of Sequence Pattern

One young and one middle-aged adult recognized a repeating pattern and both were able to repeat half of the sequence correctly. In the older adults group, one participant was able to correctly repeat the whole sequence and four participants were able to correctly repeat half of the sequence. Four older participants reported the impression of a pattern but could not repeat the sequence. Although we realize that verbal reports can fail to reveal explicit knowledge, especially when knowledge is held with low confidence, we decided to further investigate whether potential sequence awareness of these nine older participants may have affected performance on the SRTT, at least on an exploratory basis. Therefore, the group of older adults was divided into two subgroups (awareness vs. no awareness). For motor sequence learning, a mixed-design ANOVA with *sequence awareness* (awareness vs. no awareness), *time* (baseline vs. EoA) and *condition* (sequence vs. random) was conducted and the *condition* by* sequence awareness* interaction tended to be significant [*F*_(1,16)_ = 3.34; *p* = 0.08; $\eta_{p}^{2}$ = 0.17]. While RTs in sequence and random trials in older adults with no sequence awareness did not differ significantly from each other (sequence mean = 658.01 ± 55.33 [SD]; random mean = 670.44 ± 48.56; *p* = 0.52), older adults with sequence awareness were significantly faster in sequence than in random trials (sequence mean = 609.92 ± 53.26; random mean = 679.95 ± 39.55; *t*_(6)_ = 2.63; *p =* 0.04). No other effects involving the factor *sequence awareness* were significant (all *p* > 0.43). Due to small subgroup sample sizes, we performed additional nonparametric Wilcoxon signed-rank tests comparing sequence and random trials in subgroups with sequence awareness and no sequence awareness. These analyses revealed similar results as parametric analyses.

Regarding early susceptibility to interference and consolidation, mixed-design ANOVAs with *time* (early interference: EoA_S_ vs. SInterference; consolidation: SInterference vs. sequence consolidation; EoA_S_ vs. sequence consolidation) and *sequence awareness* as factors did not yield significant effects involving the factor *sequence awareness* (all *p* > 0.11).

### WM Performance

**Figure 5:**
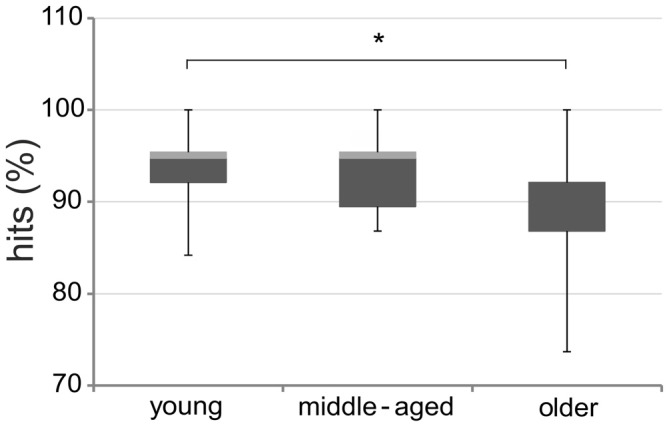
**Performance on 2-back tasks.** Percentage of hits on the 2-back tasks in young, middle-aged, and older adults. Boxplots indicate medians (horizontal line between dark gray and light gray box) and the 75th (top of box) and 25th (bottom of box) percentile ranges; the whiskers indicate full ranges. Note that visuospatial and verbal data were pooled. *p < 0.05.

Kruskal-Wallis tests revealed no significant performance differences between groups for 0- and 1-back tasks (all *p* > 0.65). However, we found significant differences between groups for the 2-back task (χ^2^ = 7.55; *df* = 2; *p* = 0.02; Figure 5). *Post hoc* comparisons based upon *critical mean rank differences* (Schaich and Hamerle, 1984) revealed that older adults performed significantly worse than young adults (*p* < 0.05), while no significant difference between young and middle-aged or middle-aged and older adults emerged (all *p* > 0.05).

### Relationship Between SRTT and WM Performance

To assess a possible relationship between motor sequence learning and WM performance, learning-related difference scores were correlated with percentages of hits in 1- and 2-back tasks (pooled for verbal and visuospatial subtypes) in each age group as well as in the entire group. No significant correlation emerged (all *p* > 0.08)*.* However, baseline RTs on the SRTT and percentage of hits in 2-back tasks were negatively correlated when all participants were included in the analysis (*ρ* = −0.47; *p* < 0.001) indicating that faster baseline RTs were associated with better WM performance.

## Discussion

The present study aimed at investigating whether implicit motor sequence learning changes across the adult life span and whether age-related differences might vary with WM performance. Sixty volunteers aged between 18 and 71 years completed the SRTT as well as verbal and visuospatial *n*-back tasks. As a main result, we found motor sequence learning in young and older but not in middle-aged adults. The largest gain in RT was found in the elderly, but their baseline RT was significantly slower as compared to the younger groups. Interestingly, in older adults, training on the SRTT yielded RTs comparable to that of middle-aged adults. Young and older adults showed interference of the newly learned motor sequence. After 1 h, this susceptibility to interference was reduced indicating motor sequence consolidation even in older participants. Off-line improvement was observed only in young and middle-aged adults. Importantly, this improvement was not specific to sequence trials indicating unspecific RT facilitation. Although motor sequence learning was not significantly related to WM performance regardless of age, the data indicate reduced WM abilities in older as compared to younger adults when WM load was high. Moreover, baseline RTs were found to significantly vary with WM across age groups.

### Implicit Motor Sequence Learning Across the Adult Life Span

The present data suggest that implicit motor sequence learning changes across the adult life span. Although young adults showed faster RTs than older ones during baseline already, both age groups significantly improved RTs after training on the SRTT. As RTs decreased for sequentially but not for randomly presented bars, this gain reflects the acquisition of sequence-specific knowledge rather than general, unspecific RT facilitation. Furthermore, older adults showed greater RT improvement than young ones. Immediately after learning, both young and older adults were susceptible to interference. Even though findings of age-related changes in motor sequence learning are by far not consistent, the present data are in line with numerous studies reporting no decline (Howard and Howard, 1992; Janacsek and Nemeth, 2012) or even better motor sequence learning in the elderly when compared to younger adults (Brown et al., 2009). Furthermore, age-related deficits in motor sequence learning are often only observed when task demands are high like learning of complex sequences or dual-tasking (Frensch and Miner, 1994; Howard et al., 2004). Also, when compared to subjects of studies reporting age-related motor sequence learning deficits (mean age of around 70; Frensch and Miner, 1994; Howard et al., 2004), our group of older adults (mean age of 60.20 ± 1.50) was relatively young.

Surprisingly, middle-aged adults between the age of 30 and 50 failed to show motor sequence learning. The literature of age-related changes in motor sequence learning has focused on the comparison between young and older adults. Two studies investigated implicit motor sequence learning across the adult life span (Gaillard et al., 2009; Janacsek et al., 2012). Gaillard et al. (2009) examined groups with an age range similar to the one in the present study on the SRTT, but they failed to find significant differences when comparing middle-aged to young and older adults, respectively. In contrast, Janacsek et al. (2012) reported a decline in sequence learning in participants above the age of 44. Although this is in accordance with the present data, the authors also found learning deficits in older adults (Janacsek et al., 2012). Interestingly, when asking participants about potential sequence awareness in the present study, only one middle-aged and one young adult were aware of the repeating pattern of the task, whereas nine older participants perceived a sequential pattern or were even able to repeat at least parts of the sequence. Although the assessment of sequence awareness in the present study was not optimal, results nevertheless might led to the speculation that the elderly may adopt explicit rather than implicit learning strategies. This interpretation is supported by the observation that older adults with potential sequence awareness tended to be faster in sequence than in random trials. It is thus conceivable that the lack of motor sequence learning in middle-aged but not in older adults in the present study might be at least partly attributable to a stronger involvement of a compensatory explicit strategy in the elderly. In line with this, a neuroimaging study reported greater activity in MTL areas (Dennis and Cabeza, 2011)—which have been related to explicit learning (Cohen et al., 1985, 1999)—during the SRTT in adults above the age of 60 as compared to younger ones. Moreover, results of the middle-aged participants led to the speculation that at this age a transition may occur in which implicit learning may become less effective while compensatory strategies have not been successfully adopted, yet.

### Consolidation and Unspecific RT Facilitation Across the Adult Life Span

To our knowledge, this is the first study investigating both susceptibility to interference and off-line improvement as two components of consolidation across the adult life span. As outlined above, young and older adults showed susceptibility to interference immediately after learning which was reduced over the off-line period indicating motor sequence consolidation in these age groups. These findings support the hypothesis of the occurrence of consolidation manifesting as stabilization of skill between sessions in older adults (Brown et al., 2009). In terms of sequence-specific off-line improvement, the second component of motor sequence consolidation, a different pattern of results emerged. Both young and middle-aged adults exhibited gains in RT over the off-line period possibly subserved by neuroplastic changes induced by the motor training (for review, see Dayan and Cohen, 2011). However, as indicated by the non-significant three-way interaction between *time, condition* and *group*, the observed RT facilitation was not specific to sequence trials. Although this finding is in line with a general RT facilitation but lacking sequence-specific improvement over off-line periods on the related alternating serial reaction task (ASRT) reported by Nemeth and Janacsek (2011), it contradicts previous results of sequence-specific off-line improvement on the SRTT (Robertson et al., 2004b; Brown et al., 2009). However, it is important to keep in mind that these authors implemented off-line periods of 12–24 h. Thus, the break of 1 h in the present study might have been too short to allow sequence-specific RT improvements.

In contrast to young and middle-aged adults, older adults failed to exhibit further gains in RT over the off-line period which replicates previous results (Brown et al., 2009; Nemeth and Janacsek, 2011) and suggests that off-line RT facilitation is affected by aging. Previous studies revealed a decrease in motor cortical plasticity in older adults when compared to younger ones (Sawaki et al., 2003; Rogasch et al., 2009; Fathi et al., 2010). Thus, one might speculate that such changes might at least partly account for lacking off-line gains in older adults in the present study. Interestingly, reduced susceptibility to interference even in the elderly reveals evidence for the assumption that different processes may underlie off-line improvement and reduced interference, although both are assumed to reflect consolidation. But, we realize that the present data do not allow any conclusions regarding brain processes underlying the observed behavioral effects.

### WM Performance and Its Relation to Motor Sequence Learning

We found age-related differences in WM independent of task subtype as assessed by the *n*-back tasks. Older participants were impaired when WM load was high replicating the results of former studies (Mattay et al., 2006; Geerligs et al., 2014). Mattay et al. (2006) showed that this age-related decline was associated with reduced DLPFC activation in older as compared to young adults.

The present data did not provide evidence for a significant association between implicit motor sequence learning and WM performance in any of the examined groups. Results of a previous study suggest that WM might be stronger related to explicit than implicit learning (Unsworth and Engle, 2005). Yet, recent studies provided first evidence for an association between implicit motor sequence learning and WM capacity in young as well as in older adults (Bo et al., 2011, 2012). It should be stressed, however, that they used a change detection task in which participants were required to detect changes between sample and test displays. This type of WM task rather taps into storage capacity of WM ability (Luck and Vogel, 1997; Fukuda et al., 2010) whereas *n*-back tasks involve a strong updating component (Braver et al., 1997; Rottschy et al., 2012). Thus, conflicting results might be at least partly due to differences in mental processes required to perform the respective tasks.

Although there was no significant relationship between learning scores and WM performance, baseline RT on the SRTT was inversely correlated with WM performance when task difficulty was high (i.e., high WM load). It is thus conceivable that although WM is not significantly associated with motor sequence learning, poorer WM performance might account for considerably slower baseline RTs, e.g., as observed in older participants.

### Limitations

A major limitation of the present study refers to the assessment of sequence awareness. We realize that computerized tasks such as the process dissociation procedure (Destrebecqz and Cleeremans, 2001) or recognition tasks (Shanks and Johnstone, 1999) constitute more sensitive tests of explicit knowledge. Thus, the present investigation of sequence awareness has to be considered as rather exploratory. Nevertheless, we think that the observation that almost half of the older participants perceived (or were even able to repeat) a sequential pattern might be a hint that with advancing age, different strategies might come into play to accomplish motor sequence learning. In future studies, these processes should be assessed and differentiated more specifically. Moreover, the sample size—especially with regard to sequence awareness subgroup analysis—was small. Thus, the results have to be interpreted with caution, even though it is conceivable that some of the older adults in the present study learned the sequence by compensatory explicit strategies. Furthermore, although we did not assess motivation before, during or after the experiment, higher motivation in older subjects may account for the observed behavioral effects as well.

Finally, to keep verbal and visuospatial *n*-back tasks as comparable as possible, we used only capital letters during verbal *n*-back tasks. Although this is common practice, we are not able to completely rule out the possibility that participants used the shape of the stimuli rather than verbal encoding.

## Conclusion

The present data indicate changes in implicit motor sequence learning across the adult life span. Middle-aged adults failed to show motor sequence learning while older adults exhibited gains that were even greater than that of young adults, possibly by adopting explicit rather than implicit learning. The observation of reduced susceptibility to interference 1 h after initial training in young and older adults suggests that consolidation also occurs in the elderly. However, older adults—in contrast to young and middle-aged adults—did not show further (unspecific) off-line gains. As previously shown, older participants showed poorer WM performance than young adults when WM load was high; but, WM processes assessed by *n*-back tasks seem to be unrelated to motor sequence learning independent of age.

## Author Contributions

SNM: conception and design of the experiment, data collection and analysis, interpretation of the data, drafting the article; AK: interpretation of the data, critical revision of the article; MS: interpretation of the data, critical revision of the article; BP: conception and design of the experiment, interpretation of the data, critical revision of the article.

## Conflict of Interest Statement

The authors declare that the research was conducted in the absence of any commercial or financial relationships that could be construed as a potential conflict of interest.
